# Does more rehabilitation lead to better upper limb outcomes after stroke? A systematic review

**DOI:** 10.3389/fresc.2026.1753677

**Published:** 2026-03-02

**Authors:** Manush Patel, Ines Serrada, Brenton Hordacre

**Affiliations:** Innovation, IMPlementation and Clinical Translation (IIMPACT) in Health, College of Health, Adelaide University, Adelaide, SA, Australia

**Keywords:** chronic, dosage, rehabilitation, stroke, subacute, therapy, upper-limb

## Abstract

**Purpose:**

To determine whether increased rehabilitation duration improved upper-limb outcomes in people with subacute and chronic stroke. Where possible, we explored evidence for dose-response relationship or a ceiling effect for recovery.

**Methods:**

A systematic review was conducted by searching Medline, EMBASE, EMCARE and PsycInfo. Randomised trials delivering the same type of upper-limb rehabilitation, but with a > 50% greater duration in one group (high dose) compared to another (low dose) were included. Risk of bias was assessed using the Cochrane risk of bias tool. Certainty of evidence was assessed using GRADE. Meta-analyses explored effects of increased rehabilitation duration and subgroup analyses explored a 50%–100%, 100%–150% and ≥200% increases in dose.

**Results:**

Four studies (*n* = 151) were included. Fugl-Meyer Assessment data were available from a single multi-arm trial, from which two intervention-control comparisons were derived based on therapy dose (*n* = 25), demonstrating greater rehabilitation duration led to improvement (*p* = 0.009). Meta-analyses including three studies (*n* = 89) found and a non-significant effect on the Action Research Arm Test (*p* = 0.266), both with a low certainty of evidence. Subgroup analyses showed that smaller dose increases (50%–150%) had limited benefits but doses ≥200% led to significant improvements in upper-limb function on the Action Research Arm Test (MD = 13.86, 95% CI: 3.43 to 24.29; *p* = 0.009).

**Conclusion:**

Greater rehabilitation duration can lead to better upper-limb outcomes; however, an increase of at least 200% in duration might be required.

**Systematic Review Registration:**

PROSPERO CRD42024604104.

## 1 Introduction

Stroke is an acute, focal neurological deficit resulting from vascular pathology of the central nervous system. Globally, it affects 15 million people each year, of which seven million die, and another five million are left with long-term disability (1). As the third leading cause of death and disability combined (1), stroke imposes a major global burden. From 1990 to 2017, the number of stroke events increased by 76%, resulting in a loss of 132 million disability-adjusted life years (2). Approximately 40% of people experience persistent upper-limb dysfunction post-stroke (3). Impaired upper-limb recovery limits an individual's ability to engage in activities of daily living and can negatively impact quality of life and mental health (4, 5). Despite its importance, upper-limb rehabilitation is often underemphasised in clinical practice (6). Serrada et al. found that in the acute phase of stroke recovery, only eight minutes of rehabilitation were dedicated to upper-limb, highlighting the under-delivery of targeted therapy (7). Qualitative studies show that patients consider upper-limb recovery a priority yet see it as a neglected aspect of rehabilitation (8). The prioritisation becomes more obvious when individuals are discharged from the hospital and faced with challenges of self-care at home and community participation (3).

Recovery of upper-limb function following a stroke is influenced by the timing, type and amount of rehabilitation. The early post-stroke period is characterised rapid improvement within the first months. This is thought to occur through spontaneous biological recovery (including resolution of oedema, reperfusion of penumbral tissue, and reversal of diaschisis) and experience-dependent changes in brain organisation (9), commonly referred to as neuroplasticity. However, evidence suggests that substantial improvements can occur with increased therapy dose, even in the chronic phase, outside the period of heightened biological recovery. For instance, a recent randomised trial demonstrated 90 h of intensive therapy over 5 weeks, led to large gains in upper limb recovery and quality of life compared to usual care (10). Furthermore, a randomised controlled trial, in which all groups received different types but same duration of therapy (300 h over 12 weeks), found that chronic stroke survivors (>1-year post-stroke) achieved clinically meaningful improvements with a mean of 11.5 points on the Fugl-Meyer Assessment (FMA) (11). Finally, observational data showed that 68.3% of participants receiving 90 h of therapy over three weeks surpassed the minimal clinically important difference (MCID) of 5.25 points on the FMA at the six-month follow-up (12). These findings suggest that dose may play an important role, particularly when delivered in high amounts; however, the absence of controlled groups (of lower dose) is a limitation of these studies.

Several systematic reviews have examined the dose-response relationship in stroke rehabilitation, with most finding that increased therapy duration correlates with improved functional outcomes (13–19). However, many reviews have focused on acute or early subacute phases where there are different biological mechanisms influencing neuroplasticity and recovery as opposed to late subacute and chronic stages of recovery (13–15, 17, 18). Furthermore, others have not controlled for therapy content which could potentially confound ability to isolate the effect of dose alone (13, 16, 17, 19). There is need to systematically evaluate the evidence for greater rehabilitation dose and upper-limb outcomes in people with late subacute and chronic stroke, while ensuring content (type) of therapy remains consistent. Understanding this is clinically relevant as upper-limb rehabilitation often becomes a priority after hospital discharge and evidence is needed to guide community rehabilitation.

Therefore, the primary objective of this review was to determine whether higher amounts of upper-limb rehabilitation improve outcomes in people with late subacute or chronic stroke. Secondary objectives were to explore whether there was evidence for a dose-response relationship or if there was a ceiling effect, whereby the continued increase in amount of rehabilitation led to no evidence of further improvement.

## 2 Methods

### 2.1 Protocol

This review adhered to the updated criteria of Preferred Reporting Items for Systematic Reviews and Meta-Analyses 2020 (PRISMA 2020) (20). The protocol was pre-registered on PROSPERO on 21st October 2024 (registration number CRD42024604104).

### 2.2 Search strategy

A pilot search was initially conducted on Medline and EMBASE to assess the feasibility of this review by confirming the availability of potentially relevant studies. The search also yielded no results for recent systematic reviews exploring the specific aims of this review. Therefore, an electronic search was conducted on Medline, EMBASE, EMCARE and PsycINFO via Ovid from inception of databases to 17th December 2024, for relevant studies available in English. Validity of the search was confirmed by using several pre-identified relevant articles. Full search strategy can be found in Supplementary Appendix A1.

### 2.3 Inclusion criteria

Included studies were required to be randomised controlled trials of upper-limb rehabilitation in adults (≥18 years) with a diagnosis of stroke, in the late subacute (>3–6 months) or chronic (>6 months) phase of recovery (21). Interventions were required to have a minimum of two groups delivering different therapy durations; a low-dose group (considered the control) and a high-dose group (>50% increase in duration). Dose was defined as the total amount of practice, operationalised as the time spent in upper-limb therapy (hours). Studies were required to have the same therapy content between groups. Included studies were required to have a measure for upper-limb outcomes, such as the Fugl-Meyer Assessment (FMA), the Action Research Arm Test (ARAT) and the Wolf Motor Function Test (WMFT). The measures were required to be performed pre- and post-interventions.

### 2.4 Exclusion criteria

Theses, non-traditional outputs (portfolios, creative works and research projects from external bodies), conference abstracts and studies where full text could not be located were excluded. Additionally, studies were excluded if they compared usual care with usual care plus another therapy without controlling for therapy content, compared therapy with no therapy or with a sham control and did not report the therapy duration (e.g., total hours).

### 2.5 Study selection

Following the search, all references were imported to EndNote where duplicates were removed. The remaining records were imported to Covidence, where further duplicates were automatically removed by the automation tool. Two independent reviewers screened titles and abstracts for relevance, with the screening distributed among three reviewers. All studies excluded by the automation tool were cross-checked by one reviewer to confirm ineligibility. Two reviewers then independently reviewed full texts of the remaining studies to screen for potentially eligible studies. At all stages, any conflicts and discrepancies were resolved through a discussion and involved a third reviewer where necessary.

### 2.6 Data extraction

One reviewer extracted all the relevant data from each study which was then cross-checked by a second reviewer. Extracted information included, first author's name and publication year, study design, participant's—age, sex, sample size enrolled and completed, time since stroke, description and duration of interventions and upper-limb outcome data. Where key data such as sample size, mean values, or standard deviations for outcome measures was missing, study authors were contacted via email. Where required, contact was attempted on up to two occasions, separated by at least two weeks.

### 2.7 Risk of bias assessment

Two independent reviewers used the Cochrane risk of bias tool—two (RoB2) to assess bias of the included studies (22). The tool measures bias across five areas: bias from the randomisation process, deviations from intended interventions, missing outcome data, measuring of outcome and the selection of reported results. Each study was assigned an overall risk of bias rating (low risk, some concerns, high risk) based on these domains. Summary graphs and tables were showcased with the robvis tool.

### 2.8 Data synthesis

A narrative synthesis was conducted for all the included studies. Meta-analyses were conducted to summarise and compare results where outcomes could be pooled. The meta-analyses examined the effect of increased upper-limb rehabilitation duration on upper-limb recovery post-stroke to calculate pooled effect sizes. To address the primary objective, the pre-and post-intervention values from the primary (FMA) and secondary (ARAT, WMFT) outcome measures were used to explore the effect of increased rehabilitation duration. Effect sizes were reported as mean difference (MD) with a 95% confidence interval (CI). A sensitivity analysis was conducted if significant heterogeneity was present. Certainty of evidence was rated as high, moderate, low or very low using GRADE methodology (23). GRADE considers risk of bias (informed by RoB2), inconsistency, indirectness, imprecision and publication bias. To investigate secondary objectives, secondary analyses were conducted involving subgroups. The subgroup analyses were done by groupings based on percentage increase (50%–100%, 100%–150% and ≥200%) of therapy dose.

## 3 Results

### 3.1 Search results

**Figure 1:**
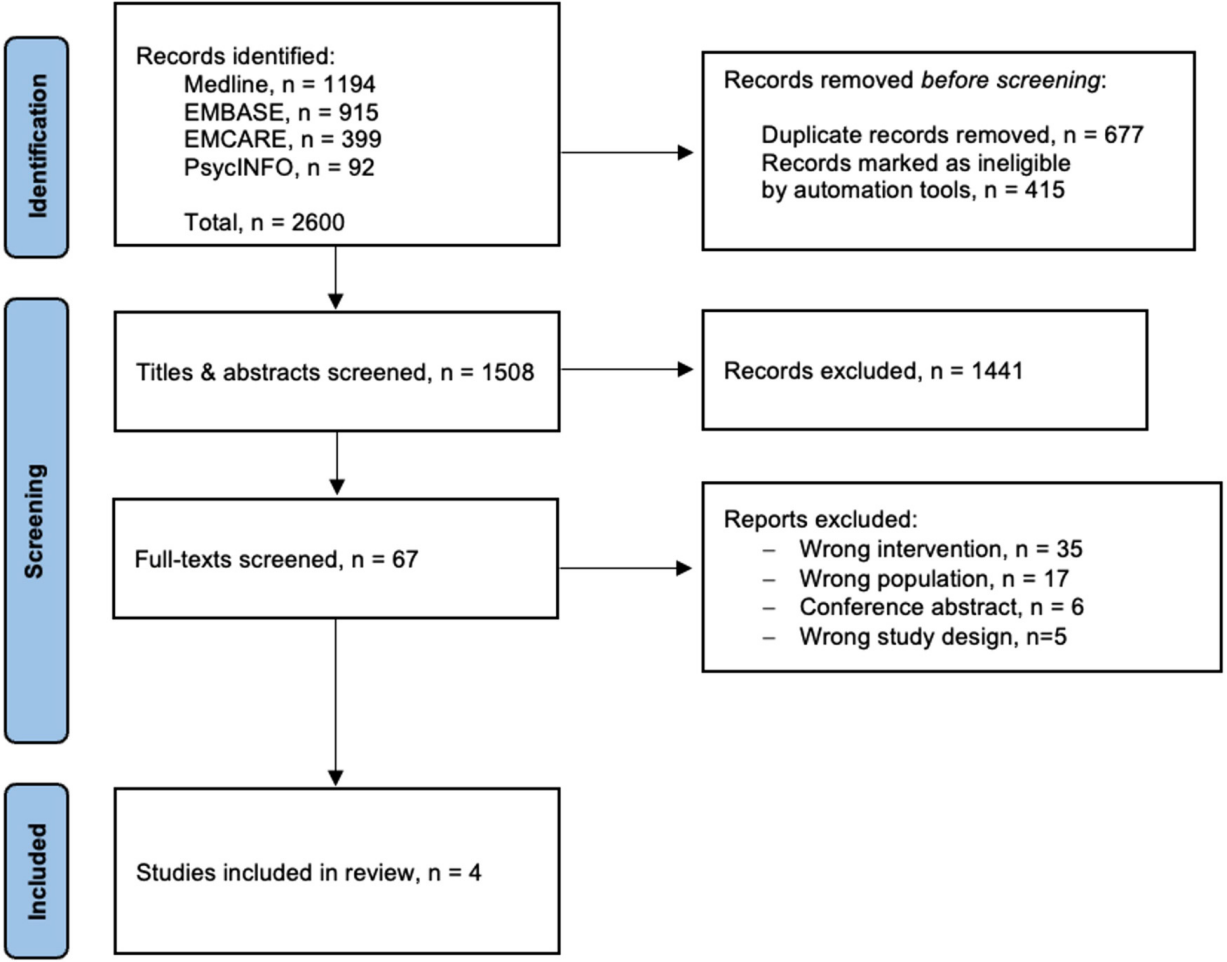
PRISMA flow diagram.

The electronic search identified 2,600 studies across all the databases, after screening, four studies were included in the review (Figure 1).

### 3.2 Study characteristics

Four studies included a total of 151 participants (24–27). The mean age across studies ranged from 57 to 61 years and included roughly 57% males and 43% females. Most participants (*n* = 148) were in the chronic phase of stroke recovery (>6 months), with only three participants in the late subacute phase (>3 months) from one study (24). All studies delivered upper-limb therapy, with a minimum of one group receiving a higher duration of therapy and a lower duration group of the same therapy. Total time spent in rehabilitation ranged from 15 to 80 h, delivered across three to five sessions per week for six to ten weeks. Three studies included multiple treatment-dosage groups that met the inclusion criteria (25–27). All studies used the ARAT to assess upper-limb function, with two also reporting on the FMA and one on the WMFT. One study could not be included in the meta-analyses as essential data (standard deviations) were not available and could not be retrieved from the author (25). A full summary of study characteristics is provided (Table 1).

**Table 1:** Summary of patient demographics and intervention details.

Author	Study Design	High Dose group (*n* =)	Low Dose Group (*n*=)	Age (Years)	Time Since Stroke (Range, Months), [Mean]	Intervention Group (Duration, % Increase)	Control Group (Duration)	Outcome Measure
Ross et al. (24)	RCT	5	5	57 (SD 14)	3–55.4 [29.8]	45 h/6 wk (200%)	15 h/6 wk	ARAT, WMFT Timing = 0, 6 wk
Page et al. (25)	RCT	7	8	61 (SD 12.3)	NR [53.8]	45 h/10 wk (80%)	25 h/10 wk	ARAT, FMA Timing = 0, 10 wk
Page et al. (26)	RCT	16	9	58 (SD 10)	NR [36]	Group A: 40 h/8 wk (100%) Group B: 80 h/8 wk (300%)	20 h/8 wk	ARAT, FMA Timing = 0, 8 wk
Lang et al. (27)	RCT	35	19	60 (SD 11.7)	6–221 [NR]	Group A: 25.3 h/8 wk (93.38%) Group B: 32.8 h/8 wk (141.18%)	13.6 h/8 wk	ARAT Timing = 0, 8 wk

NR, Not Reported in the study; h, hours; wk, weeks; RCT, randomised control trial; SD, standard deviation; UL, upper-limb; ARAT, Action Research Arm Test; WMFT, Wolf motor function test; FMA, Fugl-Meyer Assessment, Group (A and B) denote different intervention groups, Values are mean unless otherwise specified.

### 3.3 Narrative synthesis of results

Ross et al. included two groups receiving 15 h and 45 h of task-specific upper-limb therapy (24). The 45-hour group had a mean change of 12.4 ± 20.7 on the ARAT and 13.4 ± 19 on the WMFT compared to the 15-hour, with mean change of 2.6 ± 5.8 (ARAT) and 0.4 ± 1 (WMFT) post-intervention. Page et al. (26) explored three groups receiving 20, 40, 80 h of repetitive task-specific upper-limb therapy (26). The 80-hour group showed a mean ARAT change of 3.7 ± 3.0 and FMA change of 4.1 ± 2.9; the 40-hour group changed by 1.4 ± 1.8 (ARAT) and 1.3 ± 2.2 (FMA); the 20-hour group changed 1.9 ± 3.9 (ARAT) and 1.9 ± 1.6 (FMA). Lang et al. compared three groups 13.6, 26.3 and 32.8 h of task-specific upper-limb therapy (27). The ARAT scores changed from 33.7 ± 7.9 to 37.8 ± 8.8 in the 13.6- hour group, from 32.1 ± 12.3 to 35.7 ± 14.3 in the 26.3-hour group and from 31.6 ± 10.3 to 36.9 ± 12.6 in the 32.8-hour group. Page et al. included two groups receiving 25 and 45 h of repetitive task-specific upper-limb therapy (25). The 25-hour group improved scores from 24.22 to 26.78 on the ARAT and FMA scores from 30.86 to 34.20. The 45-hour group improved from 25.17 to 26.78 on the ARAT and 28.33 to 34.00 on the FMA.

### 3.4 Risk of bias

**Figure 2:**
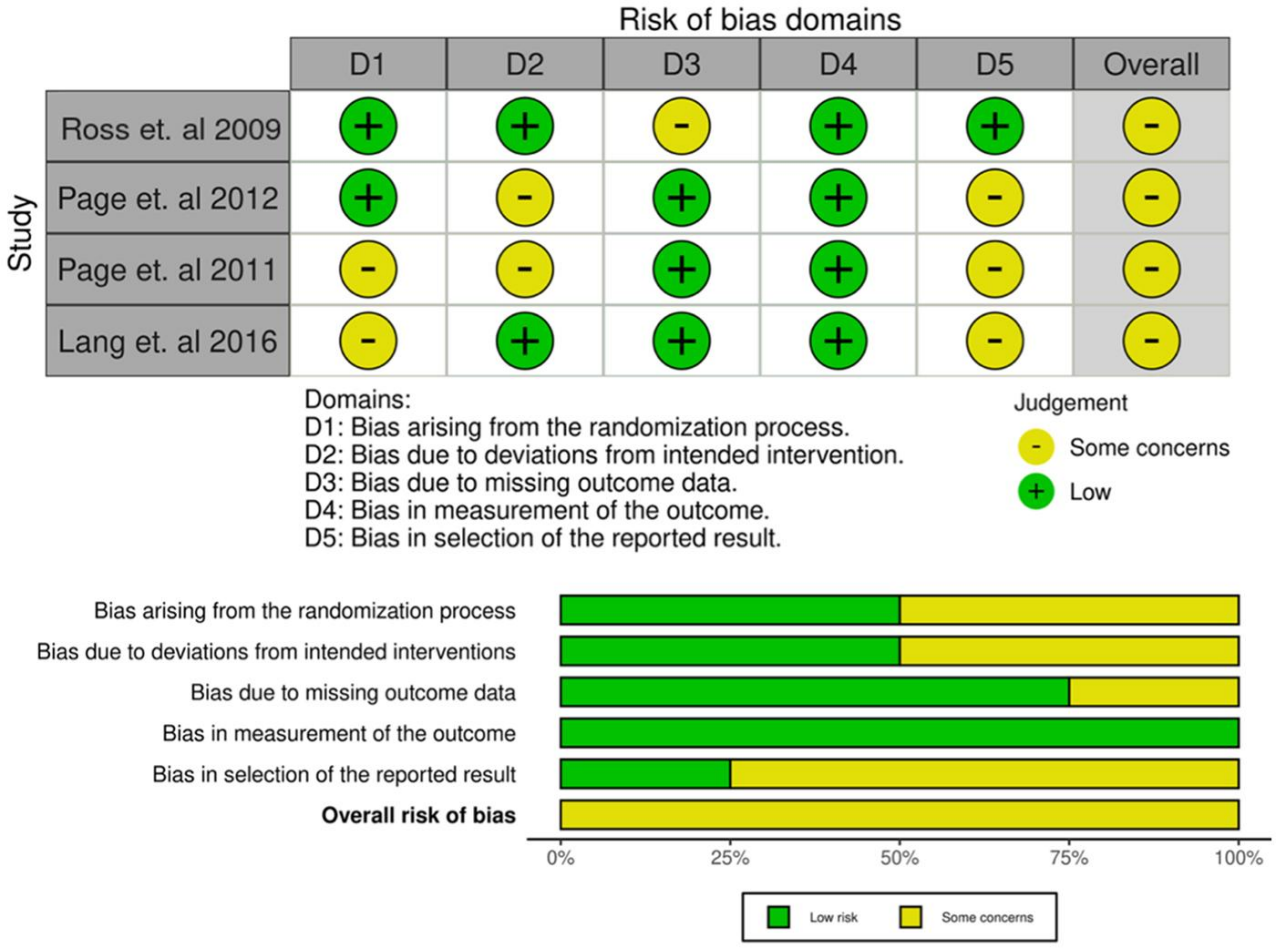
Risk of bias summary and graph for all studies.

Two studies had low risk in the randomisation process, while the other two studies showed “some concerns” due to inadequate allocation concealment (24–27). Bias due to deviations from intended intervention was low in two studies and raised “some concerns” in the others due to lack of blinding. Three studies had low risk for missing outcome data. All studies had low risk for bias in outcome measurement. Overall, all studies were rated as having “some concerns” (Figure 2).

### 3.5 Meta-analyses results

#### 3.5.1 Primary analysis: effect of increased upper-limb rehabilitation duration on upper-limb recovery

Analysis 1.1 Fugl-Meyer Assessment

**Figure 3:**
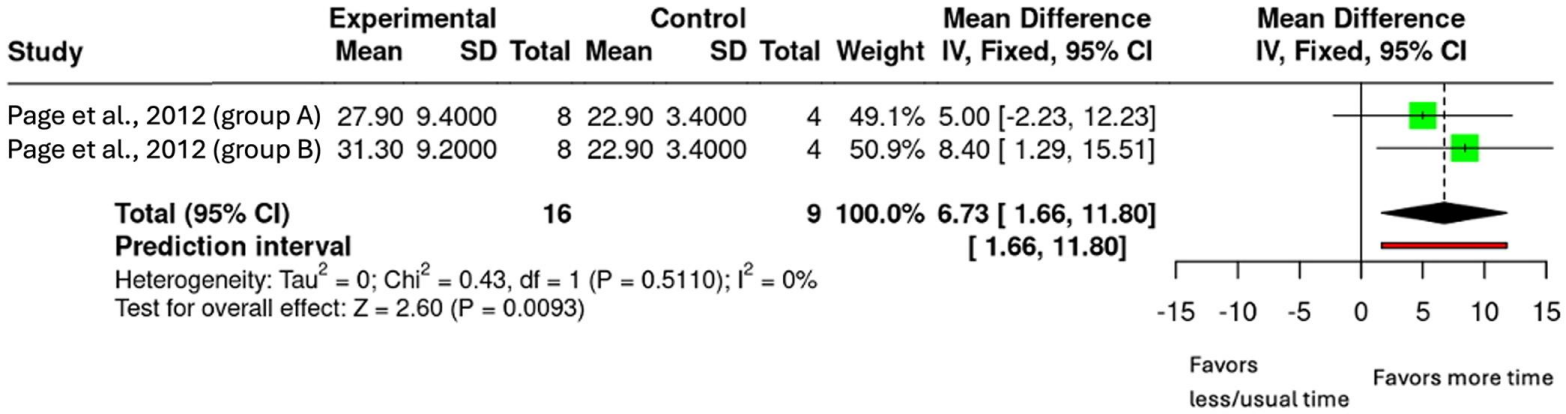
Mean difference in FMA scores by upper-limb rehabilitation duration.

One study contributed two intervention arms in the analysis with FMA outcomes comparing different upper-limb rehabilitation durations (*n* = 25, Figure 3) (26). The pooled mean difference was 6.73 (95% CI: 1.66 to 11.80; *p* = 0.009). Certainty of evidence (GRADE) was assessed as low, downgraded once for risk of bias (studies had some concerns) and once for imprecision (small sample size and wide confidence interval).

Analysis 1.2 Action Research Arm Test

**Figure 4:**
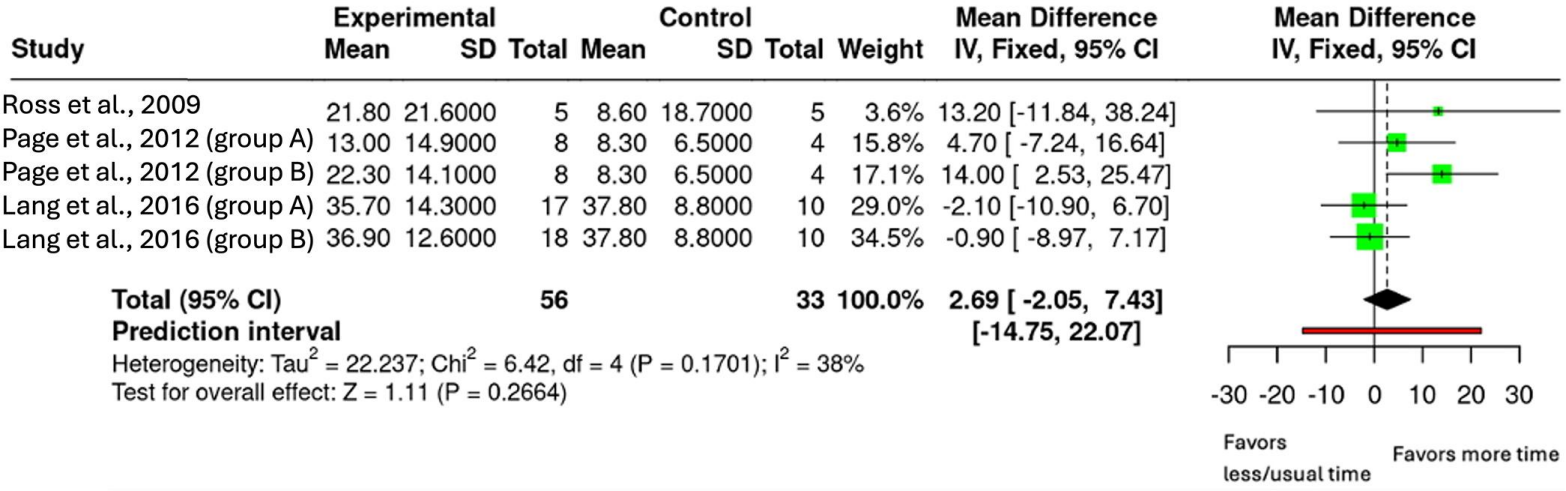
Mean difference in ARAT scores by upper-limb rehabilitation duration.

Data from three studies were included to investigate the effect of increased upper-limb rehabilitation duration on ARAT scores (*n* = 89, Figure 4) (24, 26, 27). Two studies contributed two intervention arms each as they had multiple intervention groups that met the inclusion criteria, resulting in five comparisons (26, 27). The pooled analysis showed a mean difference of 2.69 (95% CI: −2.05 to 7.43; *p* = 0.266), indicating no statistically significant effect of increased rehabilitation duration on ARAT outcomes. Heterogeneity was moderate (I^2^ = 38%). Certainty of evidence (GRADE) was assessed as low, downgraded once for risk of bias (studies had some concerns) and once for imprecision (small sample size).

#### 3.5.2 Subgroup analyses

Analysis 2.1 50%–100% Additional Therapy on ARAT

**Figure 5:**
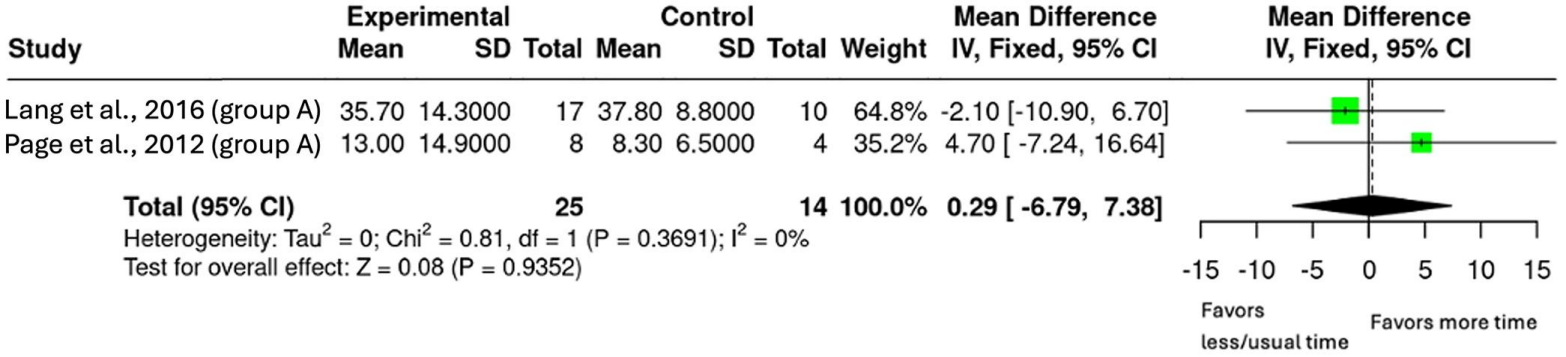
Mean difference in ARAT scores with 50%–100% additional upper-limb therapy.

Two intervention arms from two studies where the increase in upper-limb therapy dose was between 50% and 100% were included (*n* = 39, Figure 5) (26, 27). The pooled mean difference was 0.29 (95% CI: −6.79 to 7.38; *p* *=* 0.935).

Analysis 2.2 100%–150% Additional Therapy on ARAT.

**Figure 6:**
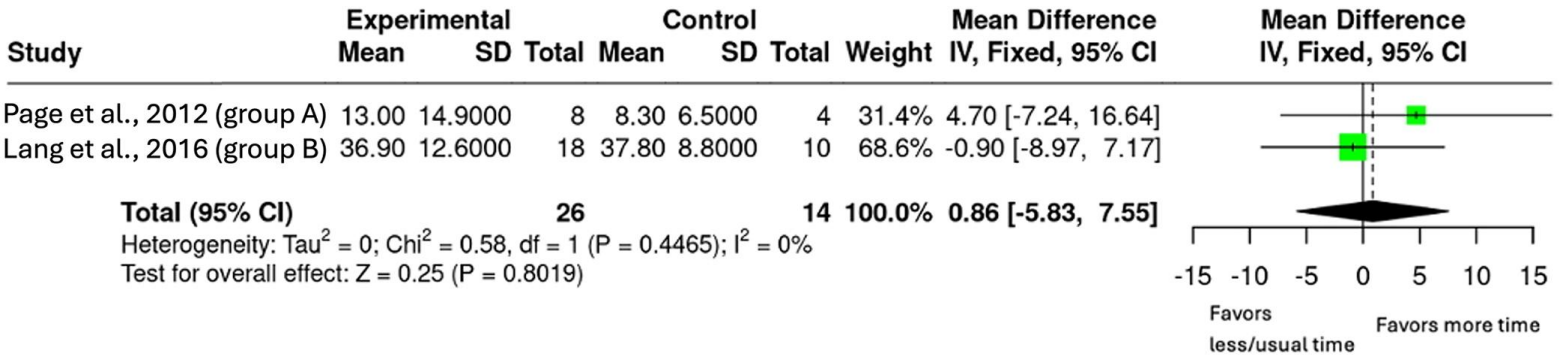
Mean difference in ARAT scores with 100%-150% additional upper-limb therapy.

Two studies had intervention arms where the dose increase for the intervention group was between 100%–150% (*n* = 40, Figure 6) (26, 27). The pooled mean difference was 0.89 (95% CI: −5.83 to 7.55; *p* = 0.801), suggesting no significant increase on the ARAT.

Analysis 2.3 ≥ 200% Additional Therapy on ARAT.

**Figure 7:**
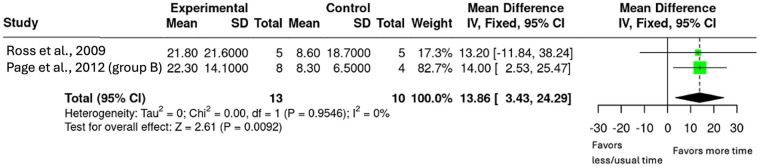
Mean difference in ARAT scores with ≥200% additional upper-limb therapy.

The largest therapy doses in the intervention groups came from two studies (*n* = 23, Figure 7) (24, 26). This subgroup included a 200% increase in therapy dose from Ross et al. and a 300% increase from Page et al. (26) (24, 26). The pooled mean difference was 13.86 (95% CI: 3.43–24.29; *p* = 0.009).

## 4 Discussion

This review aimed to explore whether increasing the duration of upper-limb rehabilitation by 50% or more leads to better outcomes, with secondary aims to explore dose-response relationships or potential ceiling effects. The limited available data showed that increasing therapy duration led to a greater change on the FMA but with low certainty of evidence. Effects were smaller for the ARAT, also with a low certainty of evidence. Across all the included studies, the highest dose group (>200% increase) showed the largest improvements.

Across all four studies, a consistent trend emerged where higher doses of upper-limb therapy were associated with bigger improvements in motor outcomes. In Ross et al., the 45-hour group had substantially larger gains in both ARAT and WMFT compared to the lower dose group (15-hour) (24). Page et al. (26) also found a similar result where the 80-hour group achieved the largest gains on the ARAT and FMA compared to the lowest dose group (20-hour) (26). In Page et al. (25), the 45-hour group showed slightly greater FMA gains, while ARAT gains were similar across both high and low dose groups (25). Lang et al., while having lowest overall therapy doses, reported small but consistent ARAT improvements across all three groups, with the largest mean gain (5.3 points) seen in the highest dose group (32.8 h) (27). Overall, these findings support a positive association between therapy dose and upper-limb recovery, with greater gains generally observed at or above 45 h of therapy.

The meta-analyses addressing the primary objective of this review found evidence on the FMA data from Page et al. (26) (26). The pooled analysis showed a mean difference of 6.73 (95% CI: 1.66–11.80; *p* = 0.009), exceeding the MCID of 5.25 for chronic stroke (28). Looking individually at the intervention arms, the highest dose group (80 h of therapy, 300%) achieved the MCID while the lower dose group (40 h of therapy, 100%) did not, suggesting that a substantial increase in therapy dose is needed to see meaningful gains (26). This aligns with previous trial data (10), and observational findings (12), that reported that intensive therapy led to gains that far exceeded MCID values. For the ARAT, the effect was not significant, but the direction of effect was in favour of the intervention group across three studies (24, 26, 27). The pooled mean difference was 2.69 (95% CI: −2.05 to 7.43; *p* = 0.266), meaning MCID was not achieved. Interestingly, previous review by Cooke et al. (14) which focused on the acute phase of stroke recovery where neuroplasticity is enhanced, reported a similar mean difference of 2.2 (95% CI: −6.0,10.4). These findings suggest that increased upper-limb therapy can still improve upper-limb outcomes, even in the later stages of stroke recovery where neuroplasticity may be reduced.

The secondary objectives exploring the evidence of dose-response relationship or if there is a ceiling effect were addressed via subgroup analyses. The subgroups were based on percentage increase in therapy duration (50%–100%, 100%–150%, and ≥200%). The results showed that smaller doses (50%–150%) produced minimal effects however, when the dose increased to ≥200%, there were significant and clinically meaningful outcomes on the ARAT with a mean difference of 13.86 (95% CI: 3.43–24.29, *p* = 0.009). Notably, both groups in the ≥200% increase surpassed the MCID of 5.7 (24, 26, 29). While the mean difference increased progressively (0.29, 0.86 and 13.86 respectively), the pattern did not appear to be linear and there was no evidence of a ceiling effect with limited studies available. These findings are similar to the review by Schneider et al. which found that a 240% increase in therapy was required to see guaranteed upper-limb improvements using the receiver-operator characteristic (ROC) curve (18). Importantly, the review by Schneider et al. included studies that were mostly in the acute and early subacute phases where neuroplasticity is enhanced (18). Finding similar dose results from this review, focused on the late subacute and chronic phase of stroke recovery, provides evidence that increased rehabilitation can still drive meaningful upper-limb outcomes, even in the later stages of stroke recovery.

Altogether, the findings from this review suggest that while increased therapy duration can lead to better upper-limb outcomes post-stroke, a substantial dose (≥200%) may be required to achieve meaningful improvements in the late subacute and chronic phases of stroke recovery. These results support the notion of dose-response relationship without clear evidence for a ceiling effect from the limited number of studies.

### 4.1 Limitations

This review is limited by the small number of included studies, with only three eligible for meta-analyses. Furthermore, intervention protocols, outcome measures and participant characteristics varied substantially across studies. Although upper-limb activity was consistently measured using the ARAT across all studies, only one study reported impairment-level outcomes using the FMA and another using the WMFT for activity measure. The limited number of studies reporting these impairment-level measures precluded pooled analysis at that level. While the overall findings suggest that more therapy is associated with better outcomes on the FMA, and that a ≥ 200% increase is needed to see larger results on the ARAT, more studies are needed to provide robust evidence to verify this. Furthermore, timing of intervention should be considered. While all included studies included people in the sub-acute and chronic phase, we note time since stroke varied between studies. It may be that even greater amounts of rehabilitation are needed in those who are very chronic. Another limitation was that some potentially relevant studies used different intervention types across dose groups. They could not be included as the type of intervention may influence the outcome. To rigorously explore the effect of dose, more studies that control the type of intervention across different doses are needed.

### 4.2 Implications for practice and future research

Across the four included studies, the low-dose (considered the control) group consistently provided upper-limb therapy between 13.6 and 25 h over six to ten weeks. This relative consistency can allow a practical extrapolation: a minimum 200% increase would equate to roughly 40–75 h of therapy over six to ten weeks. The two highest dose arms that met or exceeded this dose showed clinically meaningful results in upper-limb recovery post-stroke (24, 26). This suggests that future research and clinical guidelines could consider this as a potential benchmark for achieving functional gains in the late subacute and chronic stroke population.

There is a need for large-scale, high-quality RCTs with multiple upper-limb outcome measures, transparent trial protocols in the late subacute and chronic phase of stroke recovery and specifically looking at upper-limb recovery. This will assist in investigating threshold effects and identify patient subgroups most likely to benefit from an increased dose of rehabilitation.

## 5 Conclusion

This review found that increasing upper-limb rehabilitation can lead to improved upper-limb outcomes in the late subacute and chronic phase of stroke recovery. Clinically meaningful results on the FMA support the benefits of higher therapy dose. The ARAT results showed a non-significant effect however, the direction of effect was in favour of more therapy time. Subgroup analyses suggest a ≥ 200% increase in dose may be required to see meaningful gains on the ARAT. No evidence was found on the ceiling effect with the limited number of studies.

## Data Availability

The original contributions presented in the study are included in the article/Supplementary Material, further inquiries can be directed to the corresponding author.
