# Supplementary material for: Does more rehabilitation lead to better upper limb outcomes after stroke? A systematic review
Source: Front Rehabil Sci. 2026 Mar 2;7:1753677. doi: 10.3389/fresc.2026.1753677 (PMC12989500; doi:10.3389/fresc.2026.1753677)
Supplement: Supplementary file 1 [file Table1.docx]

Supplementary Material

# Appendices

Appendix 1 – Medline search strategy

| 1. | exp cerebrovascular disorders/ or stroke/ |
| --- | --- |
| 2. | (stroke* or poststroke or post-stroke or chronic stroke or chronic-stroke or sub-acute stroke).ti,ab,kf. |
| 3. | 1 or 2 |
| 4. | exp Rehabilitation/ |
| 5. | (rehabilitat* or recovery*).ti,ab,kf. |
| 6. | 4 or 5 |
| 7. | exp Physical Therapy Modalities/ |
| 8. | Occupational Therapy/ |
| 9. | Physiotherapy.mp. |
| 10. | 7 or 8 or 9 |
| 11. | exp Upper Extremity/ |
| 12. | ("upper extremit*" or "upper limb*" or "upper body").ti,ab,kf. |
| 13. | 11 or 12 |
| 14. | dos$.mp. |
| 15. | intensit$.mp. |
| 16. | total hour$.mp. |
| 17. | amount$.mp. |
| 18. | time$.mp. |
| 19. | duration$.mp. |
| 20. | 14 or 15 or 16 or 17 or 18 or 19 |
| 21. | 3 and 6 and 10 and 13 and 20 |

**
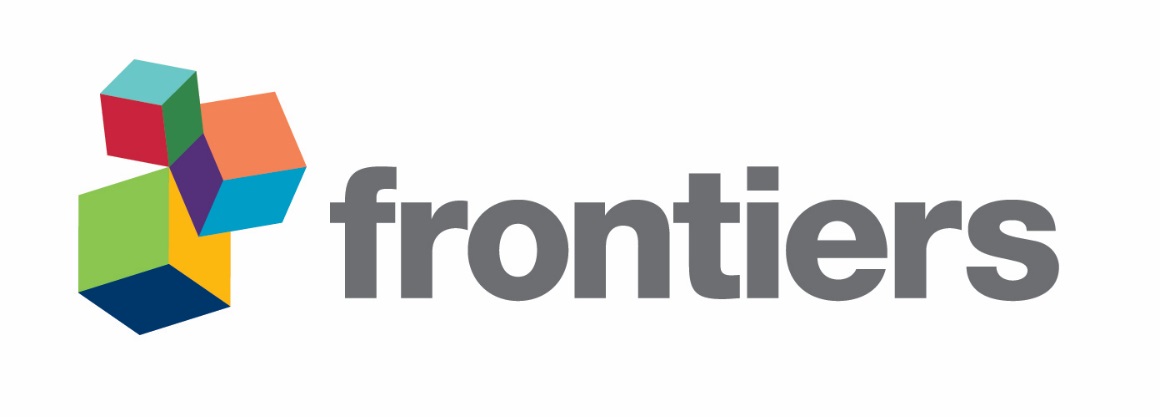
**
